# A Comprehensive Narrative Review of Neuropathic Pain: From Pathophysiology to Surgical Treatment

**DOI:** 10.7759/cureus.58025

**Published:** 2024-04-11

**Authors:** Bruno Lima Pessôa, Wilhelmina N Hauwanga, Anusha Thomas, Gabriella Valentim, Billy McBenedict

**Affiliations:** 1 Neurosurgery, Fluminense Federal University, Niterói, BRA; 2 Family Medicine, Faculty of Medicine, Federal University of the State of Rio de Janeiro, Rio de Janeiro, BRA; 3 Neurology, Christian Medical College, Ludhiana, IND; 4 School of Nursing, Fluminense Federal University, Niterói, BRA

**Keywords:** deep brain stimulation, optogenetics, spinal cord stimulation, evidence-based medicine, chronic pain

## Abstract

Neuropathic pain is a challenging condition. Despite the immense progress made in the pathophysiology and treatment of such conditions, so much work still has to be done. New frontiers previously unexplored are now objects of study with exciting results, mainly regarding neuromodulation and optogenetics. This review explores the already known pathophysiology and the clinical and surgical treatment in the light of evidence-based medicine. Additionally, new concepts and insights are discussed, presenting the hope for the development of new paradigms in the treatment of neuropathic pain.

## Introduction and background

Neuropathic pain (NP) arises from dysfunctions or lesions that impact the somatosensory nervous system, either centrally or in the periphery. This defect in the transmission system occurs when there is a loss of input to the nervous system, resulting in various negative sensory symptoms [1]. Numerous factors can contribute to NP, including conditions like polyneuropathy, diabetes, chemotherapy, herpes zoster infection, chronic alcohol abuse, small fiber neuropathy, trigeminal pain, and post-stroke pain. Typically, patients experience sensations such as tingling, shooting pain, and spontaneous or evoked discomfort following either harmful or non-harmful stimuli [1]. While significant advancements have been achieved in functional examinations, like functional magnetic resonance imaging (fMRI), enhancing our comprehension of NP, diagnosis predominantly remains clinical. This underscores the pivotal role of thorough history-taking and physical examination in accurately identifying the etiology of NP (see Table 1, Appendices) [2]. The management of NP encompasses drug therapies, surgical interventions, and an interdisciplinary approach. Recognized as one of the most complex fields in medicine, NP has remained contentious, and its treatment remains challenging despite advancements. However, significant progress has been made in understanding its pathophysiology, leading to advancements in medical and surgical treatments, including emerging techniques like optogenetics. Despite the diverse mechanisms of action associated with each treatment modality-whether clinical, surgical, or optogenetics-they all culminate in altering neural circuits, influencing neuronal firing patterns and communication. This highlights the pivotal importance of gaining a deeper understanding of the pathophysiology of NP, which will ultimately facilitate the development of more targeted and mechanism-based treatment approaches [3].

The scarcity of randomized controlled trials (RCTs) and robust evidence for many NP treatments is widely acknowledged. However, the absence of conclusive evidence does not necessarily render treatment ineffective. This realization has led to the emergence of the concept of science-based medicine (SBM) alongside evidence-based medicine (EBM) over the past decade [4,5]. Science-based medicine (SBM) offers insight into fundamental treatment principles, mechanisms, and efficacy without the stringent regulations and ethical considerations inherent in evidence-based medicine (EBM), notably randomized controlled trials (RCTs). While SBM acknowledges RCTs as the gold standard for minimizing bias in clinical trials, it recognizes that alternative methods exist to establish treatment plausibility. SBM's versatility becomes especially crucial for treatments where conducting clinical trials poses ethical challenges, suggesting a potentially significant role for SBM in the future [4,5]. Patients' quality of life is significantly diminished by NP, impeding daily activities and compromising mental well-being. Its relentless nature restricts mobility, challenges routine tasks, and induces feelings of frustration and helplessness. Moreover, NP often triggers anxiety, depression, and mood disturbances, creating a vicious cycle of pain and psychological distress [1]. This comprehensive narrative review aims to provide a detailed overview of NP, covering its pathophysiology, clinical manifestations, diagnostic approaches, and treatment modalities including surgical interventions. By synthesizing the existing literature and evidence, this review sought to elucidate the complex mechanisms underlying NP and explore the role of surgical treatments in managing this challenging condition.

## Review

Pathophysiology 

The comprehension of NP has advanced significantly, owing to the development of animal models replicating clinical pain syndromes. Particularly noteworthy are experiments dating back to the 1970s, wherein injury inflicted on peripheral nerves revealed distinct pathophysiological mechanisms compared to those triggered by acute noxious stimuli. Consequently, much of our current understanding of the science of pain stems from basic science research [6]. The signs and symptoms observed in NP cannot be attributed to a single mechanism alone. This complexity may explain why relying solely on one treatment modality often proves ineffective. Embracing a mechanism-based approach to treatment holds promise for achieving more effective pain control, as it addresses the multifaceted nature of NP [1]. With this in mind, a pressing question emerges: Can we apply findings from basic research conducted on animal models to enhance our understanding of human conditions? While this notion remains contentious, the author advocates for the integration of such information between basic and clinical research endeavors.

**Figure 1:**
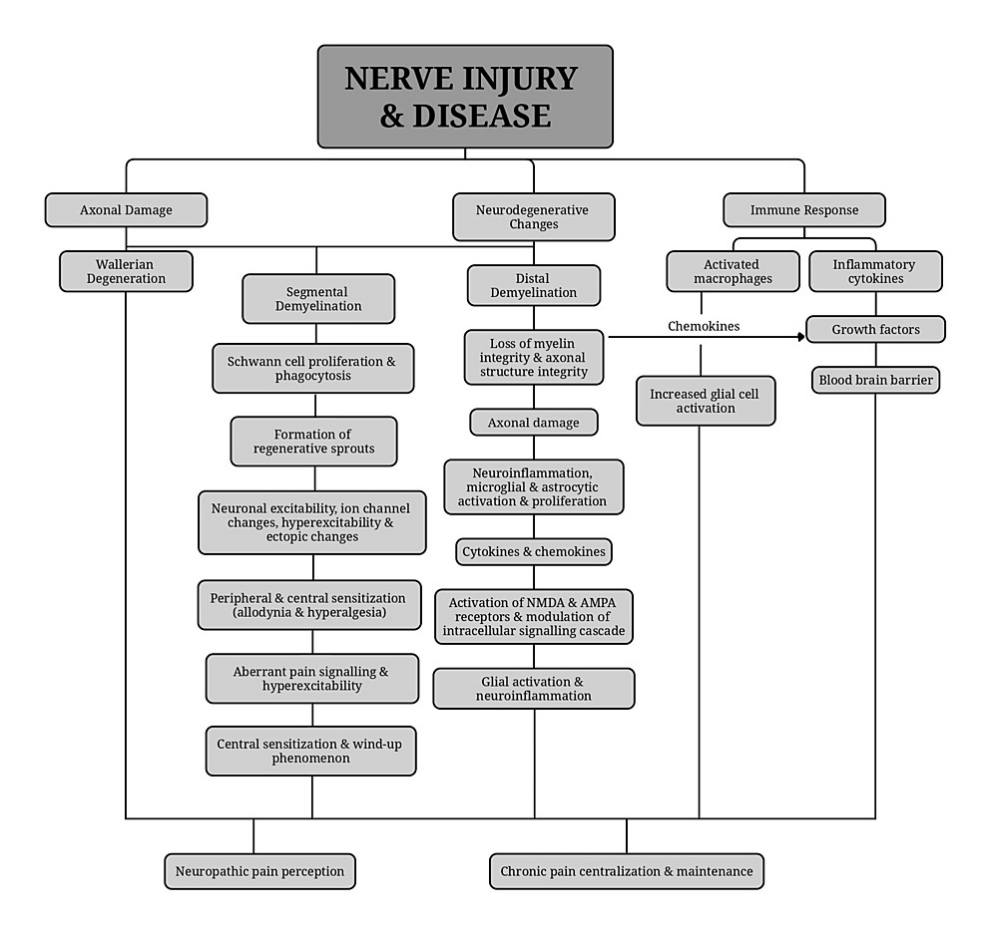
The pathophysiology of neuropathic pain NMDA: N-methyl-D-aspartate receptors; AMPA: α-amino-3-hydroxy-5-methyl-4-isoxazolepropionic acid receptors. The image is prepared by the authors.

As technology advances, certain tools have emerged as crucial assets in clinical research. These include functional imaging, quantitative sensory testing (QST), laser-evoked potentials (LEPs), and contact heat-evoked potentials (CHEPs). These tools offer invaluable insights into concepts that have previously eluded us. Furthermore, they have the potential to elucidate individual pain mechanisms, paving the way for more tailored treatments, specifically, mechanism-based treatments, instead of causative-based treatment [7]. It's important to note that NP typically arises from lesions affecting the afferent pathways, a principle supported by both clinical research and basic science. Moreover, various diseases may exhibit similar mechanisms underlying NP. Furthermore, NP can be influenced by diverse mechanisms, leading to either single or multiple symptoms [7]. Currently, many aspects of the pathophysiology of NP remain elusive, despite the elucidation of underlying mechanisms for some of its symptoms. Below, we outline some of these mechanisms as shown in Figure 1.

Ectopic nerve activity and peripheral mechanism

C fibers and A delta fibers respond to various stimuli, including mechanical, thermal, and chemical insults. However, in individuals with NP, ectopic nerve activity (ENA) occurs not only within the injured nerve itself but also in adjacent nociceptive afferents that remain non-injured [7]. This phenomenon has been documented through microneurography across various conditions, including phantom limb pain, diabetic neuropathy, and erythromelalgia. Microneurography reveals the spontaneous nerve activity of nociceptive afferents in these patients, attributed to channelopathy. This condition lowers the action potential threshold, heightens membrane excitability, and subsequently leads to ectopic nerve activity (ENA). Such activity can spontaneously induce sensations like tingling and paroxysmal shooting pain, even in the absence of tissue damage. Addressing this activity through local anesthetic blockade, such as lidocaine administration at a stump or in cases of complex regional pain syndrome (CRPS) type I, may alleviate pain, allodynia, and signs of autonomic dysfunction [8-10]. 

Similarly, in certain conditions like erythromelalgia, there's an upregulation of voltage-gated sodium channels observed in both healthy and injured nerve fibers. This upregulation lowers the action potential threshold, leading to ectopic nerve activity (ENA). This mechanism is also evident in central lesions, where similar changes affect second-order nociceptive neurons [7]. Additionally, Rowbotham and Fields delved into the impact of afferent lesions and pain manifestation in post-herpetic neuralgia (PHN). Their discussion centered on the hypothesis of synaptic remodeling within C-fibers, proposing that the severity of pain correlates with the preservation of thermal sensory function rather than its loss. In essence, they suggest that allodynia in PHN stems from abnormal activity in preserved small-diameter primary afferent nociceptors. Consequently, the reversal of this phenomenon could be achieved through peripheral C fiber blockade [11]. Furthermore, the authors propose that the activity within primary afferents, particularly sensitized C-nociceptors, in response to an external stimulus, might be accountable for initiating and sustaining various spontaneous and evoked pains. This assertion finds support in experiments where local anesthetics were applied to the painful region, resulting in significant pain relief [11].

In addition to those theories, over the last decade, has emerged the discussion about the role of both immune and glial cells in the pathophysiology of NP. From this standpoint the neurons would not be the only cell or mechanism involved in genesis and maintenance of NP, changing the way that we used to think about the pathophysiology of NP. Doing so, new paradigms in the treatment may be achieved, allowing us to modulate the immunological system after a nerve injury, as well as the alterations in glial cells, instead of targeting only the suppression of neuronal activity after a nerve injury [12]. Thus, according to this theory, nerve lesions occur as a consequence of reactions within the nerve itself, the dorsal root ganglion (DRG), and the spinal cord. Macrophages, T lymphocytes, and mast cells are recruited and activated in these sites, leading to the proliferation of Schwann cells. These cells create a matrix for future regenerating axons. As a result, prominent microglial activation occurs in the aforementioned sites [12].

Altogether, microglia and Schwann cells may contribute to Wallerian degeneration distal to a nerve injury, as well as to the release of neurotrophic pro-inflammatory cytokines, such as tumor necrosis factor α (TNF α). The overexpression of microglia within the CNS will sustain NP through the continuous release of immune modulators. All these reactions will result in the hypersensitivity of nociceptors and modulation of sensory neuron gene expression [12]. That being said, what if we were able to target the treatment of NP at molecular and genetic levels, using immunosuppressive drugs or those that could intervene with glial functions? What if we could halt the process whereby intact nociceptors (without underlying nerve damage) undergo ectopic activation and suffer from pathological receptor expression?

Central sensitization

Such a mechanism occurs under two conditions: ectopic activity in afferent fibers, with the loss of inhibitory GABAergic interneurons in the spinal cord, and injury within the central nervous system (at spinal or supraspinal levels). This injury might result in a reduction of the influence of analgesic descending pathways originating in the brainstem, leading to worsened pain due to disinhibition. These processes explain why we find allodynia and hyperalgesia not only in territories restricted to the receptive field of the injured nerve itself but also in adjacent areas where the nerve is intact or uninjured [7]. As well described by Moore et al., partial peripheral nerve injuries are accompanied by selective loss of GABAergic inhibition in the superficial dorsal horn of the spinal cord. In contrast, all excitatory amino acids and neuropeptides within the dorsal horn of the spinal cord continue to play their roles, leading to the overexpression of voltage-gated sodium channels [13]. This, in turn, will favor postsynaptic changes in second-order nociceptive neurons, culminating in a scenario where neuronal hyperexcitability becomes the norm. Consequently, normal tactile stimuli will result in allodynia and hyperalgesia due to the overexpression of low-threshold mechanosensitive Aβ and Aδ afferent fibers, which will activate second-order nociceptive neurons, resulting in the aforementioned symptoms [7,13-15]. 

Treatment

Medical Treatment

All medical treatments applied to NP are based on the principle of modulating neuronal excitability. This entails the modulation of ion channel activity or reinforcement of the endogenous inhibitory system. However, these treatments are not tailored to target specific pain disorders or individual patients. Furthermore, the presence of several different mechanisms in NP may explain why patients respond differently to the same condition, even when they take the same drugs. Additionally, the presence of psychological problems in these patients adds another layer of complexity [7]. Therefore, tricyclic antidepressants, duloxetine, opioids, gabapentin, pregabalin, carbamazepine, lidocaine, and capsaicin are among the most commonly used medications currently (Table 2, Appendices). These drugs are supported by relatively strong evidence for the treatment of NP based on randomized controlled trials (RCTs) [7,16]. In addition, recommendations are made based on the aforementioned studies. Tricyclic antidepressants (TCAs), topical lidocaine, selective serotonin reuptake inhibitors (SSRIs), and calcium channel α2-δ ligands are considered first-line medications that have been used. However, for specific conditions such as neuropathic cancer pain, acute NP, or acute exacerbations of NP, tramadol, and opioids might also be considered first-line treatments. Additionally, some NP conditions respond better to specific drugs than others [17]. 

More recently, several other drugs have been studied clinically for NP, including acetyl-L-carnitine, alpha-lipoic acid (ALA), and cannabis. Some indications include diabetic neuropathy (acetyl-L-carnitine and alpha-lipoic acid), low back pain and sciatica (ALA), post-traumatic or post-surgical NP (cannabis), and NP related to HIV (cannabis). Despite the lack of robust evidence, these alternative treatments have been shown to be safe and well-tolerated. However, except for cannabinoids in central pain (multiple sclerosis), which have demonstrated consistent efficacy (level A of efficacy), none of the aforementioned medications have been considered first-line treatments for NP in other conditions [18,19]. Interestingly, the role of glia and immune cells in NP, rather than the exclusive role of neurons, has received more attention in recent years. This creates opportunities for the development of new drugs and treatments. Although we have not yet seen any clinical trials in this regard, targeting these mechanisms seems reasonable and logical in the opinion of the senior author. Utilizing animal models, some preclinical research has focused on the use of medications such as fluorocitrate, propentofylline, minocycline, and teriflunomide for neuroimmunomodulation [12,20]. It is believed that these drugs might act against cytokine release, thereby compromising glial metabolism. A second mechanism could involve decreasing the rate of proliferation and activity of both microglia and astrocytes, which are considered important mechanisms in sustaining NP. As a consequence, pain relief could occur [12,21]. 

Ablative Procedures

Over the last two decades, there has been a decline in the use of ablative procedures for NP. Despite their demonstrated efficacy in clinical practice and in the literature, the irreversibility of these procedures weighs against their usage. However, a few lesional surgeries are worth mentioning, such as DREZ (dorsal root entry zone) lesions, thalamotomy, and trigeminal percutaneous procedures. DREZ surgery remains the best option in treating refractory NP secondary to cervical plexus ablation, as well as an alternative treatment for complex regional pain syndrome (CRPS) and, in select cases, post-herpetic neuralgia. Although not the first choice for patients with spasticity (intrathecal baclofen being a better option), it is a worthwhile procedure for patients experiencing both NP and spasticity. 

Thalamotomy 

The thalamus stands as a critical structure within the intricate network of the brain, serving as a central hub for the transmission of information [22]. Extensively connected to the cerebral cortex via thalamocortical radiations, the thalamus assumes a paramount role in a myriad of cognitive, sensory, and executive functions [23,24]. Its profound connectivity underscores its significance in orchestrating various aspects of brain activity and behavior. Manipulation of the thalamus, such as through procedures like thalamotomy, holds the potential to exert a profound influence on both behavior and the intricate networks of the brain [25]. This makes thalamotomy a therapeutic option for a spectrum of neurological conditions, including essential tremor (ET), Parkinson’s disease (PD) [26], Holmes tremor (HT) [27], epilepsy [28], neurogenic pain [29], multiple sclerosis (MS) [30], and dyskinesia [31].

Central lateral thalamotomy targets the medial thalamus with a specific focus on the posterior part of the central lateral nucleus (CLp). This area of the thalamus holds significance as a key regulator of thalamocortical activity, playing a crucial role in sensory processing and integration [32]. Dysfunction within the CLp is implicated in NP syndromes, where it contributes to the perpetuation and amplification of low-frequency neurophysiological activity across thalamic and cortical regions. The activity within the CLp, characterized by increased theta rhythms and coherence between thalamic and cortical regions, gives rise to a phenomenon known as thalamocortical dysrhythmia, and is thought to be responsible for neurogenic pain and has been associated with chronic NP states [32]. By targeting the CLp through central lateral thalamotomy, clinicians aim to modulate and normalize thalamocortical activity, thereby alleviating symptoms associated with neurogenic pain. This approach represents a targeted intervention designed to restore neural circuitry balance and mitigate the pathological processes driving chronic pain states.

Thalamotomy can be performed using various techniques; among these techniques are radiofrequency (RFT), gamma knife radiosurgery (GKT), and magnetic resonance-guided focused ultrasound (MRgFUS). Radiofrequency thalamotomy stands as a well-established method in which an electrode is precisely placed through the brain parenchyma to deliver targeted thermal ablation to the thalamic area of interest [33]. This technique, although effective, entails an open surgical procedure, carrying inherent risks such as intracerebral hemorrhage and infection [34]. While offering the advantage of direct and precise lesioning, the invasive nature of radiofrequency thalamotomy necessitates careful consideration of associated surgical risks and postoperative management strategies.

The MRgFUS method represents a cutting-edge approach that harnesses the power of multiple extracranial ultrasonic waves to precisely target areas within the brain. By meticulously controlling the phase and amplitude of these waves under the guidance of structural and thermal images obtained via MRI, MRgFUS offers a non-invasive means of delivering therapeutic intervention directly to the intended brain region. Unlike traditional surgical approaches, MRgFUS obviates the need for skin incisions or trepanation, thereby minimizing the invasiveness of the procedure and reducing associated risks. In a multicenter clinical study, the efficacy of MRgFUS in treating tremor was evaluated and revealed a promising improvement rate of 55% on the clinical rating scale of tremor (CRST) at six months post-treatment, with sustained improvement of 56% observed after two years [35]. Despite these favorable results, certain transient side effects were noted, including sensory disturbance, taste disturbance, gait disturbance, ataxia, and dizziness [36]. However, it is important to highlight that these adverse effects were temporary in nature and largely resolved within a year following the procedure. Such findings underscore the overall safety and efficacy profile of MRgFUS as a minimally invasive approach to brain surgery, offering a promising avenue for the management of various neurological conditions.

Gamma knife radiosurgery, also known as stereotactic radiosurgery, offers a non-incisional or minimally invasive alternative to traditional open cranial surgery. Developed by Leksell, this technique relies on the precise irradiation of well-defined brain volumes, allowing for targeted ablation of pathological tissue while minimizing damage to surrounding healthy structures [37]. The GKT mechanism of action not only involves the direct ablation of the lesion but also encompasses a neuromodulatory effect on the surrounding areas. This modulation extends beyond the necrotic core to include the sub-necrotic zone, potentially contributing to a lower incidence of adverse effects such as ataxia or paraesthesia compared to other ablative techniques [33]. Furthermore, GKT offers superior accuracy compared to conventional radiofrequency thalamotomy (RFT), as it eliminates the mechanical brain shift resulting from the introduction of a rigid electrode into the intracranial space. This enhanced precision is attributed to the ability to deliver radiation with submillimeter accuracy, ensuring precise targeting of pathological tissue while sparing adjacent healthy structures [38].

GKT offers several advantages over traditional surgical techniques. One notable benefit is its transcranial approach, which virtually eliminates the risk of surgery-related infections commonly associated with invasive procedures involving the penetration of the cranial vault. Additionally, the risk of intracranial hemorrhage, which may arise from the insertion of intracranial probes in other surgical methods, is significantly reduced with GKT, enhancing overall safety and minimizing postoperative complications. Moreover, GKT enables precise control over the extent of the lesion, leading to more predictable treatment outcomes and reduced interindividual variability in therapeutic response, particularly when compared to radiofrequency thalamotomy (RFT). This heightened precision ensures targeted ablation of pathological tissue while sparing adjacent healthy structures, optimizing treatment efficacy, and minimizing the risk of unintended damage to surrounding brain regions. However, it is important to acknowledge that GKT also has its limitations. One such limitation is the delayed onset of therapeutic effects, which may take weeks or even months to manifest fully. This delay in improvement can result in lesions that are larger than intended, potentially affecting treatment outcomes and patient satisfaction. Thus, while GKT offers significant advantages in terms of safety and precision, clinicians must carefully consider the timing and expectations of treatment when utilizing this approach [39].

Young et al. conducted a study involving eight cases of essential tremor patients who underwent GKT. Their findings revealed compelling outcomes, with an impressive 87.5% rate of clinical improvement observed among the treated individuals. Specifically, 62.5% of patients experienced complete cessation of tremors, while an additional 25% reported a significant reduction in tremor severity, rendering them nearly tremor-free. However, it is noteworthy that a 12.5% failure rate was also documented in the study cohort. These results underscore the promising therapeutic potential of GKT in the management of essential tremor, highlighting its efficacy in ameliorating debilitating symptoms and improving patients' quality of life [40]. Gamma knife radiosurgery (GKT) and MR-guided focused ultrasound (MRgFUS) represent innovative non-invasive approaches for targeting specific brain regions without the necessity of skull penetration or traditional open cranial procedures [33]. While both techniques offer distinct advantages, they differ in their mechanisms and immediate effects. Gamma knife radiosurgery utilizes multiple beams of precisely focused radiation to target pathological brain areas with exceptional accuracy [33]. In contrast, MRgFUS employs focused ultrasound waves to induce thermal ablation or neuromodulation within the targeted brain tissue [41]. One notable distinction is the immediate impact of MRgFUS, which allows for real-time monitoring of lesion size and patient response during the procedure [41]. This capability enables clinicians to adjust treatment parameters promptly based on patient feedback, enhancing the precision and efficacy of the intervention.

Trigeminal Percutaneous Procedures

Trigeminal neuralgia (TN) is a debilitating condition characterized by sudden, intense, and excruciating pain within the distribution of the trigeminal nerve. The hallmark diagnostic criteria include brief paroxysms of pain triggered by seemingly innocuous stimuli such as speaking, chewing, or even light touch to the affected area, typically localized to one or more branches of the trigeminal nerve [42]. Management of TN encompasses both pharmacological and surgical interventions, with the latter reserved for cases refractory to conservative measures.

Surgical options for TN include microvascular decompression (MVD) of the trigeminal nerve root, percutaneous procedures such as radiofrequency thermocoagulation, mechanical balloon compression, and chemical neurectomy, as well as peripheral techniques and radiosurgery [43]. Each surgical approach targets different aspects of the trigeminal nerve pathway, aiming to alleviate pain and improve the patient's quality of life. However, the selection of the most appropriate surgical intervention depends on various factors, including the patient's clinical presentation, underlying etiology, comorbidities, and treatment goals. Microvascular decompression (MVD) entails the surgical repositioning of vascular structures compressing the trigeminal nerve root, thereby alleviating nerve irritation and pain. Percutaneous techniques, such as radiofrequency thermocoagulation and mechanical balloon compression, involve targeted lesioning or compression of the trigeminal nerve to disrupt aberrant pain signals. Chemical neurectomy, another percutaneous option, utilizes neurolytic agents to selectively damage nerve fibers transmitting pain impulses. Additionally, peripheral techniques and radiosurgery offer minimally invasive alternatives for patients who are not suitable candidates for conventional open surgery.

Percutaneous trigeminal rhizotomy serves as a valuable therapeutic option primarily indicated for Burchiel type 1 trigeminal neuralgia (TN), also known as typical TN. This variant of TN is characterized by recurrent, paroxysmal episodes of intense, lancinating facial pain resembling electric shocks, often triggered by innocuous stimuli [44]. These percutaneous procedures offer a minimally invasive alternative to MVD, which is particularly advantageous for patients with medical comorbidities or risk factors that preclude them from undergoing open cranial surgery. Patients who may benefit most from percutaneous trigeminal rhizotomy are those with advanced age or significant medical conditions that render them high-risk candidates for more invasive surgical interventions like MVD [45]. By providing effective pain relief with reduced surgical morbidity and shorter recovery times, percutaneous rhizotomy procedures offer a feasible treatment option for elderly patients or those with complex medical histories. Furthermore, percutaneous trigeminal rhizotomy techniques can be tailored to the individual patient's needs, allowing for customization based on factors such as pain severity, treatment response, and overall health status. This personalized approach ensures that patients receive optimal care while minimizing the potential for complications associated with more invasive surgical procedures.

Percutaneous procedures are designed to selectively target and disrupt the function of the afferent A delta and C pain nerve fibers while preserving the integrity of the A-alpha and A-beta sensory nerve fibers [43]. Microvascular decompression (MVD) is a surgical procedure aimed at alleviating the symptoms of trigeminal neuralgia by addressing the vascular loop that compresses the trigeminal nerve at its root entry zone [46]. During MVD, the offending vascular structure, often an aberrant artery or vein, is carefully identified and then gently mobilized or repositioned away from the trigeminal nerve, thus relieving the pressure and reducing irritation of the nerve fibers. MVD aims to prevent further compression and irritation of the trigeminal nerve, thereby reducing or eliminating the episodes of severe facial pain characteristic of trigeminal neuralgia. The success of MVD in providing long-term relief from trigeminal neuralgia symptoms hinges on the precise identification and decompression of the offending vascular loop. Careful intraoperative monitoring and neurophysiological testing are often employed to ensure the integrity of the trigeminal nerve and assess the efficacy of the decompression procedure.

Percutaneous balloon compression (PBC) is a minimally invasive procedure utilized in the treatment of trigeminal neuralgia, aimed at alleviating the debilitating facial pain experienced by affected individuals. This technique involves the insertion of a specialized needle through the foramen ovale, a natural opening in the skull located near the Gasserian ganglion (GG), which is a key component of the trigeminal nerve [43]. Once the needle is accurately positioned near the GG, a small balloon is then introduced through the needle and carefully inflated, exerting gentle pressure on the ganglion. This compression of the GG serves to disrupt or block the abnormal pain signals transmitted by the trigeminal nerve, effectively reducing or eliminating the episodes of intense facial pain characteristic of trigeminal neuralgia. PBC is considered an effective treatment option for patients with trigeminal neuralgia, particularly those who may not be candidates for more invasive surgical procedures such as microvascular decompression (MVD) [43]. The procedure can often be performed on an outpatient basis under local anesthesia, offering the advantages of minimal postoperative discomfort and rapid recovery.

While PBC is generally safe and well-tolerated, it is not without risks, and potential complications may include temporary numbness or weakness of the face, as well as rare but serious adverse events such as infection or injury to surrounding structures [43]. As with any medical intervention, the decision to undergo PBC should be carefully considered in consultation with a knowledgeable healthcare provider, weighing the potential benefits against the risks for each individual patient. Utilizing the Mullan technique, percutaneous balloon compression (PBC) presents a minimally invasive approach for treating trigeminal neuralgia. This technique involves precise needle placement into Meckel's cave, a small cavity within the skull housing the trigeminal ganglion. Subsequently, a Fogarty catheter is gently inserted, and the balloon is inflated with contrast material under direct radioscopic guidance. The compression of the trigeminal ganglion using the balloon, typically lasting between 1.3 and 3 minutes, results in immediate pain relief for a significant majority of patients, ranging from 80 to 90%. This rapid alleviation of symptoms underscores the efficacy of PBC as an intervention for trigeminal neuralgia [42]. In terms of long-term outcomes, PBC demonstrates sustained effectiveness, with response rates ranging from 69% to 80% even five years post-procedure [47]. This enduring relief underscores the durability of PBC as a treatment modality for trigeminal neuralgia, offering patients prolonged freedom from the debilitating pain associated with this condition.

In terms of long-term outcomes, PBC demonstrates sustained effectiveness, with response rates ranging from 69% to 80% even five years post-procedure [47]. This enduring relief underscores the durability of PBC as a treatment modality for trigeminal neuralgia, offering patients prolonged freedom from the debilitating pain associated with this condition. Among the reported complications associated with percutaneous balloon compression (PBC) for trigeminal neuralgia, masseter weakness or masticatory muscle weakness, and dysesthesias stand out as the most commonly observed adverse effects [48]. However, it is noteworthy that these complications are typically transient in nature, with resolution occurring within a span of a few weeks to months post-procedure [49]. Beyond these common complications, a spectrum of other adverse events has been documented in the literature. These include disturbances in hearing and olfaction, as well as palsies affecting cranial nerves III, IV, and VI [50]. Additionally, rare complications such as arteriovenous fistula development, meningitis, and herpes simplex labialis have been reported in isolated cases [50]. While the occurrence of these complications underscores the importance of vigilant postoperative monitoring and management, it is essential to recognize that the overall incidence of adverse events associated with PBC remains relatively low. With proper patient selection and meticulous procedural technique, the benefits of PBC in alleviating trigeminal neuralgia often outweigh the potential risks, offering patients a promising avenue for relief from their debilitating symptoms. 

Percutaneous glycerol rhizotomy (PGR) is an advantageous option for managing trigeminal neuralgia, particularly in elderly patients. The discovery of this technique by Hakanson in 1981 revolutionized treatment approaches for the condition [51]. Hakanson's original technique involved the use of a glycerol-tantalum mixture precisely injected into Meckle's cave under fluoroscopic guidance while the patient maintained an upright position with slight head flexion. Subsequently, Tan et al. further refined the procedure, demonstrating a remarkable success rate of 90.6% with modifications that allowed for the patient to be in a supine position during the intervention [52]. This evolution in technique underscores the adaptability of PGR to accommodate variations in patient positioning, providing flexibility in clinical practice. The mechanism of action behind PGR involves the introduction of glycerol into the trigeminal cistern, where it selectively damages pain-conducting nerve fibers while sparing sensory fibers. This targeted disruption of nociceptive pathways results in significant pain relief for the majority of patients undergoing PGR. 

Glycerol, known for its remarkable ability to permeate cell membranes, demonstrates unique cytoprotective properties that render it an intriguing agent in medical interventions [53]. Its low toxicity profile adds to its appeal, as it requires high doses to elicit any adverse effects. The mechanism underlying glycerol's cytoprotective action involves its ability to interact with cell membranes, facilitating its entry into cells. Once inside, glycerol exerts protective effects, shielding cells from various insults and potentially mitigating cellular damage. This property makes glycerol a valuable asset in therapeutic applications where cellular protection is paramount. Despite its favorable safety profile, caution must still be exercised when administering glycerol, particularly in higher doses. While rare, excessive doses of glycerol can lead to adverse effects. Therefore, precise dosing and careful monitoring are essential to ensuring the safety and efficacy of glycerol-based interventions. Bennett and Lunsford concluded that glycerol selectively attacks partially damaged trigeminal axons, and after their elimination, the evoked potentials appeared normalized [54]. Postoperative facial numbness is again common and typically resolves within hours to days after the operation. Varying degrees of postoperative hyperalgesia are also observed, with mild to moderate hypalgesia seen in up to 70% of cases and typically lasting no more than three to six months [55]. 

Pollock's research findings shed light on several key factors that may influence the outcomes of glycerol rhizotomy procedures for trigeminal neuralgia. Among these factors, non-constant facial pain experienced by patients prior to surgery emerges as a significant predictor of postoperative success. Patients who reported experiencing intermittent or non-continuous facial pain before undergoing glycerol injection demonstrated more favorable outcomes compared to those with constant pain. Additionally, Pollock observed that patients who experienced immediate pain upon glycerol injection during the procedure tended to achieve better results postoperatively. This immediate pain response may serve as an indicator of the responsiveness of the trigeminal nerve to the glycerol treatment, suggesting a heightened sensitivity of the affected nerve fibers. Furthermore, the development of new trigeminal deficits following the procedure was found to be associated with improved outcomes. While the occurrence of postoperative trigeminal deficits may initially raise concerns, Pollock's findings suggest that such deficits may actually signify a positive response to the glycerol rhizotomy, potentially indicating successful nerve ablation and alleviation of pain symptoms. Overall, Pollock's insights underscore the complex interplay of preoperative symptoms, intraoperative responses, and postoperative outcomes in the management of trigeminal neuralgia with glycerol rhizotomy. Understanding these factors can inform clinicians' decision-making processes and contribute to more tailored treatment approaches for patients suffering from this debilitating condition [56]. 

Radiofrequency Thermocoagulation (RFT)

The procedural setup for percutaneous glycerol rhizotomy involves meticulous attention to patient positioning and precise anatomical landmarks to guide needle insertion. The patient is positioned supine on the operating table, with specific reference points marked on the face according to Hartel's method. These markings delineate the optimal entry point for the needle, typically located approximately 3 cm lateral to the angle of the mouth. Additionally, two key reference points are identified: the medial margin of the pupil and a point 2.5 cm anterior to the tragus on the zygomatic arch. These serve as critical landmarks to guide the trajectory of the needle toward the foramen ovale. The patient usually winces or complains of pain referred to the ear when the needle is in the foramen ovale. A preliminary lesion was made by heating the electrode tip with the radiofrequency current to 70°C and maintaining it for 70 seconds. It is crucial to emphasize the importance of precision during needle cannulation to minimize the risk of complications and optimize treatment outcomes. While repeated puncture attempts may increase the likelihood of procedural complications, meticulous attention to accurate positioning can mitigate these risks and reduce the incidence of pain recurrence. Indeed, inaccurate needle placement represents a significant challenge, often leading to procedural failure and potential complications, as highlighted by Zhao et al.'s study findings [57]. Therefore, adherence to proper technique and anatomical landmarks is paramount to the success and safety of percutaneous glycerol rhizotomy procedures [57]. 

Radiofrequency rhizotomy emerges as a promising intervention, boasting an impressive initial success rate of 90%. However, the challenge lies in the recurrence of symptoms, which affect approximately 25% of cases over time. Despite this setback, the long-term efficacy remains notable, with a sustained response observed in 57.7% of patients even after five years of follow-up, as elucidated by Karol EA and Agner C in their critical review of neurosurgical interventions [47]. This dichotomy between short-term success and long-term recurrence underscores the complex nature of trigeminal neuralgia management, urging clinicians to explore adjunctive therapies or refine procedural techniques to enhance durability and minimize relapse rates [47]. Only a single reported case of intracranial hemorrhage has been documented in the literature, underscoring the rarity of this particular complication associated with the procedure [58], and meticulously documented this isolated incident, emphasizing the need for meticulous technique and careful patient selection to minimize such risks. While intracranial hemorrhage remains an uncommon outcome, its potential severity necessitates thorough preoperative evaluation and vigilant postoperative care to ensure patient safety and optimal outcomes.

Neuromodulation

The concept of neuromodulation involves a broad spectrum of alterations to the central nervous system (CNS) resulting from electrical stimulation. Despite its more recent usage in clinical practice, the history of neuromodulation dates back to as early as 1786, when Galvani described muscular contractions in frogs following an induced nerve lesion. Furthermore, in 1852, Duchenne performed electrical stimulation over the frontal branch of the facial nerve in a patient with facial nerve palsy [59,60]. Conceptually, the electrical stimulus applied to a specific nerve induces membrane changes by altering the voltage-sensitive ion channels, which in turn affects the permeability of ions in that membrane. Consequently, there may be an increase or decrease in the threshold needed to evoke an action potential. Depending on the site (target) where the stimulus is applied to the fibers, stimulation (facilitation) or inhibition of a specific task or function may occur [61].

Considered a last resort, all types of surgical intervention are only utilized when medically refractory NP has developed. That being said, one question arises: Is there sufficient evidence to support surgical interventions or any type of neuromodulation? To date, several conditions have been treated with neuromodulation with promising results, albeit with insufficient evidence. Despite the fact that some researchers have employed sham stimulation in trials on neurostimulation over the last decade, the limitations of these studies persist due to the challenges of implementing a placebo. Furthermore, some of these devices have only recently been introduced, which introduces limitations to drawing conclusions. However, it is true that level B recommendations might be found for some procedures, justifying their usage for certain pain conditions [16,62]. 

There are several neuromodulation techniques that have recently been used (Table 3, Appendices), such as spinal cord stimulation (SCS), transcutaneous electrical nerve stimulation (TENS), peripheral nerve stimulation (PNS), repetitive transcranial magnetic stimulation (rTMS), motor cortex stimulation (MCS), and deep brain stimulation (DBS). Spinal cord stimulation is a well-known technique based on gate control theory. Electrodes are implanted, surgically or percutaneously, into the epidural space, and through their discharges, there is a block of afferent stimulus when it gets to the posterior funiculi of the spinal cord, contributing to pain relief. Few conditions have been recognized with sufficient evidence to support their use, such as failed back surgery syndrome (FBSS) and complex regional pain syndrome type I (CRPS I). Both of these conditions are supported by level B recommendations [61,62]. 

In addition to these syndromes, several others have been treated with spinal cord stimulation (SCS) with positive outcomes, although evidence remains scarce. These conditions include CRPS II, peripheral nerve injury, partial spinal cord injury, diabetic neuropathy, brachial plexus lesions, and phantom pain. However, they are all categorized as class IV recommendations. Despite this, we anticipate that new indications for SCS usage will emerge in the near future, despite the lack of evidence, as the absence of evidence does not necessarily indicate a lack of clinical benefit [62]. Using superficial electrodes over the skin in the painful region, transcutaneous electrical nerve stimulation (TENS) employs low-intensity, high-frequency stimuli, activating Aβ afferents. This, in turn, induces paresthesia over the painful region and provides pain relief. TENS is a non-invasive interventional therapy, and due to its temporary effects, multiple sessions are often required. The limited availability of data regarding TENS poses challenges in drawing definitive conclusions, categorizing this procedure as level C evidence [16,62]. Utilizing a similar mechanism, albeit more invasive as it involves subcutaneous implantation in direct contact with the nerve, peripheral nerve stimulation (PNS) has garnered increased attention in recent years. Despite some promising results reported, it is currently classified as class IV [62].

Based on motor area stimulation, repetitive transcranial magnetic stimulation (rTMS) is a relatively new technique consisting of coils that connect to a pulse generator. These coils deliver electrical stimuli to the scalp, focusing on the motor area through electromagnetic induction. Consequently, this process modulates sensitive neurons via pyramidal neurons, leading to pain relief. This non-invasive method has been utilized to treat NP and major depression. Regarding NP, the evidence supporting rTMS is relatively strong (level B). Despite this, controversies exist regarding its use as a marker to identify candidates for motor cortical stimulation (MCS), although it is routinely employed [62,63]. Motor cortex stimulation (MCS), first described as a valuable alternative tool to treat NP of central origin, has been utilized since the 1990s. Its mechanism appears to be related to the reduction of activation of the hyperactive thalamus, which is typically present in central pain. The procedure involves implanting epidural electrodes over the central gyrus under general anesthesia or sedation plus local anesthesia. Several studies have correlated MCS with NP improvement, particularly in central post-stroke pain (CPSP), yielding favorable results, although no meta-analysis exists. Consequently, MCS is considered level C in patients with facial NP and CPSP [16,61,62].

Considered the most controversial surgical treatment for NP, deep brain stimulation (DBS) therapy utilizes the principle of neuromodulation, where modification of neuronal signals occurs, leading to stimulation or inhibition of certain neural networks. It is implanted in the periventricular gray matter and the sensory thalamus. Despite DBS being approved by the FDA for Parkinson’s disease, dystonia, essential tremor, and obsessive-compulsive disorder, there is a lack of evidence for its use in NP. Consequently, one meta-analysis indicates that the evidence is not strong enough to justify its usage. It appears that DBS may be more effective for nociceptive pain than for NP, although some positive results have been reported regarding pain related to amputation and facial pain. Therefore, DBS is considered class IV evidence [61,62]. 

Although there is a lack of evidence for the majority of surgical procedures in NP, one should not jump to conclusions about their inefficacy. It is worth noting that many patients who have undergone surgical procedures have reported improvements in their quality of life, even if they do not consistently report improvements in their pain levels. In other words, the improvement in pain scores does not always correlate with the improvement in quality of life. Furthermore, an unknown phenomenon is a decrease in the efficacy of neuromodulation over time. Some patients experience worsening pain as time passes. In the author’s opinion, the indications for surgical treatment in NP will increase as new targets and devices emerge.

Optogenetics

The current neuromodulation system that has been clinically used is based on Deep Brain Stimulation (DBS) and Spinal Cord Stimulation (SCS). This method stimulates neurons in a non-specific manner, affecting both neurons involved in the pathophysiology of certain diseases and those that are not. As a result, non-desirable sites are also stimulated, leading to the creation of side effects. To enhance the precision of neuromodulation, new approaches have emerged. Optogenetics, for instance, utilizes two microbial proteins known as opsins. Specifically, Chlamydomonas reinhardtii channelrhodopsin-2 (ChR2) and Natronomonas pharaonis halorhodopsin (NpHR) are employed to achieve opposite effects [3,64]. These proteins are a group of light-sensitive receptors that are naturally found in cell membranes and are coupled with G proteins. In optogenetics, these receptors can be inserted into specific neurons in the brain using stereotactic surgery, effectively turning these neurons into light receptors. This is achieved by introducing viral vectors carrying genes encoding ChR2 or NpHR into the brain. Once inside the target cells, these viral vectors integrate into the cell's genome, leading to the permanent expression of these light-sensitive proteins [3,64].

ChR2 is activated by blue light at around 470 nm, allowing Na^+^ ions to enter the cell and promoting action potential firing. In contrast, NpHR is a chloride pump activated by yellow light at 580 nm. When activated, NpHR hyperpolarizes the neuron, leading to its suppression. However, to deliver light into the brain for this process, a fiberoptic device is required. This can be achieved by inserting a microcannula, which can then photostimulate specific neurons. Ultimately, this technique allows for either stimulation or inhibition of neuronal activity, depending on the approach chosen [3]. Therefore, potential applications of optogenetics in chronic pain management could involve selectively inhibiting pain fibers using NpHR, thereby alleviating pain without affecting other types of fibers. Additionally, the function of ChR2 could be used to stimulate the endogenous analgesic system, providing pain relief [3]. However, the clinical application of optogenetics is still restricted to experimental research, and its widespread use in clinical settings is not yet realized.

## Conclusions

Significant progress has been made in the search for new pathophysiological phenomena, the development of new drugs, the use of neuromodulation, and the emerging field of optogenetics in the treatment of neuropathic pain (NP). Despite a lack of significant randomized controlled trials (RCTs) addressing the efficacy of various treatments in NP and the need for further studies, the progress achieved thus far has been substantial, resulting in a significant improvement in the quality of life of patients. Indeed, there are currently no definitive treatments for NP. However, as our understanding of the pathophysiology of pain advances, new methods may be proposed, potentially leading to greater effectiveness of various therapies for a lower percentage of patients with refractory pain. This progress could lead to a new paradigm in the treatment of chronic pain.

## Appendices

**Table 1:** Classification of neuropathic pain based on anatomy and disease *Complex Regional Pain Syndrome (CRPS). References [1,7].

Painful peripheral neuropathies	Polyneuropathies	Central pain syndromes	Mixed pain syndromes
Neuroma	Diabetes	Vascular lesions	*CRPS I and *CRPS II
Trauma	Charcot-Marie-Tooth disease	Spinal cord injury	Low back pain with radiculopathy
Phantom pain	Alcoholism	Traumatic brain injury	Cancer pain
Entrapment syndromes	Amyloidosis	Abscess	-
Diabetic neuropathic pain	Hypothyroidism	Multiple sclerosis	-
Cancer neuropathic pain	Guillain-Barré syndrome and CIDP	Syringomyelia	-
Postherpetic neuralgia	Drugs ( Anti-retrovirals, cisplatin)	Myelitis	-
Peripheral nerve tumors	Toxins (Acrylamide, arsenic, clioquinol, etc)	Parkinson's disease	-
Trigeminal neuralgia	-	-	-
Radiation plexopathy	-	-	-

**Table 2:** Evidence-based treatment for common neuropathic pain syndromes *Postherpetic neuralgia (PHN) ** Central poststroke pain (CPSP), *** Tricyclic antidepressants (TCA), ****Central Neuropathic Pain/spinal cord injury (SCI) *****extended-release (ER) [17-19].

Condition	Grade A of recommendation	Grade B of recommendation	First line
PHN*	Capsaicin 8% patch Gabapentin Gabapentin ER***** Lidocaine plasters Opioids (morphine, oxycodone, methadone) Pregabalin TCAb	Capsaicin cream Valproate	Gabapentin Pregabalin TCA*** Lidocaine plasters
Diabetic NP	Duloxetine Gabapentin-morphine TCA*** Gabapentin Nicotine agonist Nitrate derivatives Oxycodone Pregabalin TCAb Tramadol alone or with acetaminophen Venlafaxine ER*****	Botulinum toxin Dextromethorphan Gabapentin/venlafaxine Levodopa	Duloxetine Gabapentin Pregabalin TCA*** Venlafaxine ER*****
Central pain	Cannabinoids (oro-mucosal, oral) Pregabalin (SCI)	Lamotrigine (CPSP**) TCA (SCI****, CPSP**) Tramadol (SCI****) Opioids	Gabapentin Pregabalin TCA***
Trigeminal neuralgia	Carbamazepine	Oxcarbazepine	Carbamazepine Oxcarbazepine
Cancer NP	Gabapentin	Amitriptyline Tramadol	Gabapentin

**Table 3:** Summary of evidence-based surgical treatments for neuropathic pain *Transcutaneous electrical nerve stimulation (TENS) **Motor cortex stimulation (MCS) ***Repetitive transcranial magnetic stimulation (rTMS) ****Spinal cord stimulation (SCS) *****Complex Regional Pain Syndrome (CRPS) ******Postherpetic neuralgia (PHN) *******Failed back surgery syndrome (FBSS)  [16,61,62].

Condition	Procedure	Level of recommendation	Class of evidence
PHN******	TENS*	Not enough evidence	IV
Diabetic NP	TENS*	C	II
Central pain	MCS **	C	IV
-	rTMS ***	B	II
Trigeminal neuralgia	MCS **	C	IV
FBSS*******	SCS****	B	II
CRPS I*****	SCS****	B	II
CRPS II	SCS****	D	IV
Amputation pain	SCS****	D	IV
Brachial plexus damage	rTMS/ MCS	B	II

## References

[1] Jensen TS, Baron R (2003). Translation of symptoms and signs into mechanisms in neuropathic pain. Pain.

[2] de Resende MA, Nascimento OJM, Rios AAS, Quintanilha G, Ceballos LES, Araújo FP (2010). Neuropathic pain profile: the basic neurological exam of 33 patients (Article in Portuguese). Rev Bras Anestesiol.

[3] Zhang F, Aravanis AM, Adamantidis A, de Lecea L, Deisseroth K (2007). Circuit-breakers: optical technologies for probing neural signals and systems. Nat Rev Neurosci.

[4] Gorski DH, Novella SP (2014). Clinical trials of integrative medicine: testing whether magic works?. Trends Mol Med.

[5] Greenhalgh T, Howick J, Maskrey N (2014). Evidence based medicine: a movement in crisis?. BMJ.

[6] Decosterd I, Woolf CJ (2000). Spared nerve injury: an animal model of persistent peripheral neuropathic pain. Pain.

[7] Baron R, Binder A, Wasner G (2010). Neuropathic pain: diagnosis, pathophysiological mechanisms, and treatment. Lancet Neurol.

[8] Amir R, Kocsis JD, Devor M (2005). Multiple interacting sites of ectopic spike electrogenesis in primary sensory neurons. J Neurosci.

[9] Nyström B, Hagbarth KE (1981). Microelectrode recordings from transected nerves in amputees with phantom limb pain. Neurosci Lett.

[10] Orstavik K, Jørum E (2010). Microneurographic findings of relevance to pain in patients with erythromelalgia and patients with diabetic neuropathy. Neurosci Lett.

[11] Rowbotham MC, Fields HL (1996). The relationship of pain, allodynia and thermal sensation in post-herpetic neuralgia. Brain.

[12] Scholz J, Woolf CJ (2007). The neuropathic pain triad: neurons, immune cells and glia. Nat Neurosci.

[13] Moore KA, Kohno T, Karchewski LA, Scholz J, Baba H, Woolf CJ (2002). Partial peripheral nerve injury promotes a selective loss of GABAergic inhibition in the superficial dorsal horn of the spinal cord. J Neurosci.

[14] Hains BC, Saab CY, Klein JP, Craner MJ, Waxman SG (2004). Altered sodium channel expression in second-order spinal sensory neurons contributes to pain after peripheral nerve injury. J Neurosci.

[15] Finnerup NB, Jensen TS (2004). Spinal cord injury pain--mechanisms and treatment. Eur J Neurol.

[16] Finnerup NB, Otto M, McQuay HJ, Jensen TS, Sindrup SH (2005). Algorithm for neuropathic pain treatment: an evidence based proposal. Pain.

[17] Dworkin RH, O'Connor AB, Backonja M (2007). Pharmacologic management of neuropathic pain: evidence-based recommendations. Pain.

[18] Attal N, Cruccu G, Baron R, Haanpää M, Hansson P, Jensen TS, Nurmikko T (2010). EFNS guidelines on the pharmacological treatment of neuropathic pain: 2010 revision. Eur J Neurol.

[19] Schestatsky P, Vidor L, Winckler PB, Araújo TG, Caumo W (2014). Promising treatments for neuropathic pain. Arq Neuropsiquiatr.

[20] Watkins LR, Maier SF (2003). Glia: a novel drug discovery target for clinical pain. Nat Rev Drug Discov.

[21] Tawfik VL, Nutile-McMenemy N, Lacroix-Fralish ML, Deleo JA (2007). Efficacy of propentofylline, a glial modulating agent, on existing mechanical allodynia following peripheral nerve injury. Brain Behav Immun.

[22] Tomasi D, Volkow ND (2011). Association between functional connectivity hubs and brain networks. Cereb Cortex.

[23] Coenen VA, Allert N, Paus S, Kronenbürger M, Urbach H, Mädler B (2014). Modulation of the cerebello-thalamo-cortical network in thalamic deep brain stimulation for tremor: a diffusion tensor imaging study. Neurosurgery.

[24] Hwang K, Bertolero MA, Liu WB, D'Esposito M (2017). The human thalamus is an integrative hub for functional brain networks. J Neurosci.

[25] Lin SJ, Rodriguez-Rojas R, Baumeister TR (2022). Neuroimaging signatures predicting motor improvement to focused ultrasound subthalamotomy in Parkinson's disease. NPJ Parkinsons Dis.

[26] Zaaroor M, Sinai A, Goldsher D, Eran A, Nassar M, Schlesinger I (2018). Magnetic resonance-guided focused ultrasound thalamotomy for tremor: a report of 30 Parkinson's disease and essential tremor cases. J Neurosurg.

[27] Kim MC, Son BC, Miyagi Y, Kang JK (2002). Vim thalamotomy for Holmes' tremor secondary to midbrain tumour. J Neurol Neurosurg Psychiatry.

[28] Sitnikov AR, Grigoryan YA, Mishnyakova LP (2016). Bilateral radiofrequency anterior thalamotomy in intractable epilepsy patients. Zh Vopr Neirokhir Im N N Burdenko.

[29] Sarnthein J, Stern J, Aufenberg C, Rousson V, Jeanmonod D (2006). Increased EEG power and slowed dominant frequency in patients with neurogenic pain. Brain.

[30] Mathieu D, Kondziolka D, Niranjan A, Flickinger J, Lunsford LD (2007). Gamma knife thalamotomy for multiple sclerosis tremor. Surg Neurol.

[31] Lee KH (1997). MRI-guided stereotactic thalamotomy for cerebral palsy patients with mixed dyskinesia. Stereotact Funct Neurosurg.

[32] Jeanmonod D, Morel A (2009). The central lateral thalamotomy for neuropathic pain. Textbook of Stereotactic and Functional Neurosurgery.

[33] Dallapiazza RF, Lee DJ, De Vloo P (2019). Outcomes from stereotactic surgery for essential tremor. J Neurol Neurosurg Psychiatry.

[34] Fenoy AJ, Simpson RK Jr (2014). Risks of common complications in deep brain stimulation surgery: management and avoidance. J Neurosurg.

[35] Chang JW, Park CK, Lipsman N (2018). A prospective trial of magnetic resonance-guided focused ultrasound thalamotomy for essential tremor: Results at the 2-year follow-up. Ann Neurol.

[36] Meng Y, Solomon B, Boutet A (2018). Magnetic resonance-guided focused ultrasound thalamotomy for treatment of essential tremor: A 2-year outcome study. Mov Disord.

[37] Leksell L (1983). Stereotactic radiosurgery. J Neurol Neurosurg Psychiatry.

[38] Jeanmonod D, Werner B, Morel A, Michels L, Zadicario E, Schiff G, Martin E (2012). Transcranial magnetic resonance imaging-guided focused ultrasound: noninvasive central lateral thalamotomy for chronic neuropathic pain. Neurosurg Focus.

[39] Young RF, Li F, Vermeulen S, Meier R (2010). Gamma knife thalamotomy for treatment of essential tremor: long-term results. J Neurosurg.

[40] Young RF, Vermeulen SS, Grimm P, Blasko J, Posewitz A (1997). Gamma knife radiosurgery for treatment of trigeminal neuralgia: idiopathic and tumor related. Neurology.

[41] Lipsman N, Mainprize TG, Schwartz ML, Hynynen K, Lozano AM (2014). Intracranial applications of magnetic resonance-guided focused ultrasound. Neurotherapeutics.

[42] Baabor MG, Perez-Limonte L (2011). Percutaneous balloon compression of the Gasserian ganglion for the treatment of trigeminal neuralgia: personal experience of 206 patients. Advances in Minimally Invasive Surgery and Therapy for Spine and Nerves.

[43] Jones MR, Urits I, Ehrhardt KP (2019). A comprehensive review of trigeminal neuralgia. Curr Pain Headache Rep.

[44] Eller JL, Raslan AM, Burchiel KJ (2005). Trigeminal neuralgia: definition and classification. Neurosurg Focus.

[45] Cheng JS, Lim DA, Chang EF, Barbaro NM (2014). A review of percutaneous treatments for trigeminal neuralgia. Neurosurgery.

[46] Zakrzewska JM, Linskey ME (2014). Trigeminal neuralgia. BMJ.

[47] Karol EA, Agner C (1999). Technological advances in the surgical management of trigeminal neuralgia. Crit Rev Neurosurg.

[48] Lichtor T, Mullan JF (1990). A 10-year follow-up review of percutaneous microcompression of the trigeminal ganglion. J Neurosurg.

[49] Lopez BC, Hamlyn PJ, Zakrzewska JM (2004). Systematic review of ablative neurosurgical techniques for the treatment of trigeminal neuralgia. Neurosurgery.

[50] Bergenheim AT, Asplund P, Linderoth B (2013). Percutaneous retrogasserian balloon compression for trigeminal neuralgia: review of critical technical details and outcomes. World Neurosurg.

[51] Håkanson S (1981). Trigeminal neuralgia treated by the injection of glycerol into the trigeminal cistern. Neurosurgery.

[52] Tan LK, Robinson SN, Chatterjee S (1995). Glycerol versus radiofrequency rhizotomy--a comparison of their efficacy in the treatment of trigeminal neuralgia. Br J Neurosurg.

[53] Baxter DW, Schacherl U (1962). Experimental studies on the morphological changes produced by intrathecal phenol. Can Med Assoc J.

[54] Bennett MH, Lunsford LD (1984). Percutaneous retrogasserian glycerol rhizotomy for tic douloureux: Part 2. Results nd implications of trigeminal evoked potential studies. Neurosurgery.

[55] Linderoth B, Lind G (2012). Retrogasserian glycerol rhizolysis in trigeminal neuralgia. Schmidek and Sweet Operative Neurosurgical Techniques.

[56] Pollock BE (2005). Percutaneous retrogasserian glycerol rhizotomy for patients with idiopathic trigeminal neuralgia: a prospective analysis of factors related to pain relief. J Neurosurg.

[57] Zhao S, Deng M, Cai H, Meng Q, Fang W, Ke J, Long X (2018). Clinical efficacy evaluation for treating trigeminal neuralgia using a personalized digital guide plate-assisted temperature-controlled radiofrequency. J Craniofac Surg.

[58] Rath GP, Dash HH, Bithal PK, Goyal V (2009). Intracranial hemorrhage after percutaneous radiofrequency trigeminal rhizotomy. Pain Pract.

[59] Gallone P (1986). Galvani’s frog: harbinger of a new era. Electrochimica Acta.

[60] Rondot P (2005). G. B. A. Duchenne de Boulogne (1806-1875) (Article in French). J Neurol.

[61] Cukiert A, Cukiert CM (2023). Response to: dual device neuromodulation can be effective in drug resistant generalized epilepsies. Neuromodulation.

[62] Cruccu G, Aziz TZ, Garcia-Larrea L (2007). EFNS guidelines on neurostimulation therapy for neuropathic pain. Eur J Neurol.

[63] Lefaucheur JP, André-Obadia N, Antal A (2014). Evidence-based guidelines on the therapeutic use of repetitive transcranial magnetic stimulation (rTMS). Clin Neurophysiol.

[64] Tye KM, Deisseroth K (2012). Optogenetic investigation of neural circuits underlying brain disease in animal models. Nat Rev Neurosci.

